# Effect of a Physisorbed Tetrabutylammonium Cation Film on Alkaline Hydrogen Evolution Reaction on Pt Single-Crystal Electrodes

**DOI:** 10.1021/acscatal.4c01765

**Published:** 2024-05-09

**Authors:** Julia Fernández-Vidal, Marc T. M. Koper

**Affiliations:** Leiden Institute of Chemistry, Leiden University, PO Box 9502, 2300 RA Leiden, The Netherlands

**Keywords:** tetrabutylammonium, Pt single-crystal electrodes, alkaline media, hydrogen evolution reaction, site blocking, physisorbed film

## Abstract

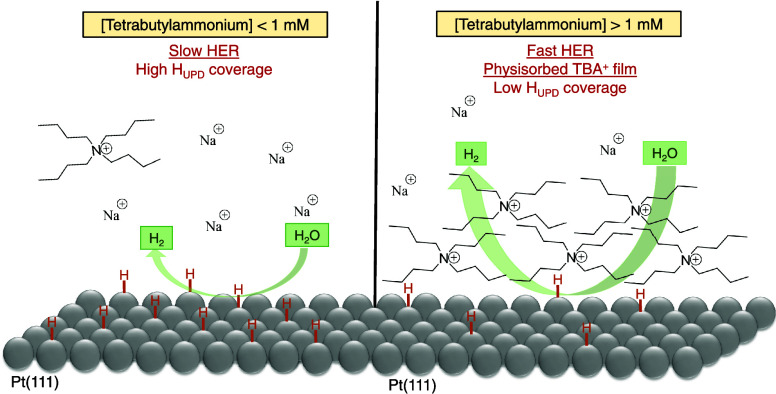

The addition of tetrabutylammonium (TBA^+^) to alkaline electrolytes enhances the hydrogen evolution reaction (HER) activity on Pt single-crystal electrodes. The concentration of TBA^+^ significantly influences the HER on Pt(111). Concentrations of ≤1 mM yield no significant effect on HER currents or the coverage of adsorbed hydrogen (H*) but exhibit an interaction with the OH_ads_ on the surface. Conversely, concentrations of >1 mM result in an apparent site-blocking effect for underpotential-deposited H* caused by the physisorption of the organic cation, which counterintuitively leads to an increase in the HER activity. The physisorption of TBA^+^ is linked to its accumulation in the diffuse layer, as it can be reversibly removed by the addition of nonadsorbing cations such as sodium. Following the previous literature on the TBA^+^ interaction with electrode surfaces, we ascribe this effect to the formation of a two-dimensional TBA^+^ film in the double layer. On stepped Pt single-crystal surfaces, TBA^+^ enhances HER activity at all concentrations, primarily at step sites. Our findings not only highlight the complexities of TBA^+^ accumulation on Pt electrodes but also offer important molecular-level insights for optimizing the HER by organic film formation on various atomic-level electrode structures.

## Introduction

The hydrogen evolution reaction (HER) is a multistep electrocatalytic process that produces hydrogen gas (H_2_). This reaction holds significant importance in reducing greenhouse gas emissions^1^ as green H_2_, generated by water electrolysis and powered by renewable energy sources, can contribute to the decarbonization of key industrial processes.^2^ Additionally, the HER is not only important in renewable energy technologies but also has played a pivotal role in the formulation of essential principles in electrocatalysis, including the Butler–Volmer equation and the Sabatier principle.^3,4^

The HER pathway is well-established, but the debate about the specific reason behind the anomalous non-Nernstian pH dependence of the HER kinetics remains,^5,6^ hindering the optimization of alkaline electrolyzers and revealing a substantial gap in our fundamental understanding of the electrochemical interface. The binding energies of the intermediates and adsorbates have traditionally been favored as activity descriptors due to their simplicity and accessibility in the rational design of electrocatalysts.^3,7^ Nevertheless, there is a growing acknowledgment that other factors influencing kinetic parameters need to be taken into account for the alkaline HER.^8−11^ The surface geometry of the catalyst,^12^ the electrolyte properties such as cation concentration and identity,^13−17^ the interfacial electrostatic potential,^18,19^ the local water structure, and the interfacial H-bond connectivity^20^ have all been linked to the pH dependence of the HER.

In particular, cations present a critical component in HER activity that has attracted scientific interest in the past decade. The arrangement of cations at the interface is influenced by the applied electrode potential and adsorbed species, leading to changes in the solvation shell, the H-bonding network, and the orientation of water molecules at the surface, which ultimately affect the accessibility of proton donors for the HER. Specifically, optimizing the interactions of the right cations with step sites in Pt-based electrodes can maximize the HER activity in alkaline conditions.^21^ In addition, it has been demonstrated that some organic molecules enhance the rate of the HER in both neutral and alkaline environments.^22,23^

Tetraalkylammonium cations (TAA^+^) are known to enhance electroreduction reactions.^24^ Recently, TAA^+^ cations have been shown to have a substantial impact on the alkaline HER kinetics on polycrystalline Pt electrodes.^25^ Compared to alkali metal cations, TAA^+^ enhances the exchange current density of alkaline HER by more than a factor of 4. The presence of TAA^+^ was suggested to increase the formation and lifetime of H-bonds among interfacial water molecules.^25,26^ This effect was linked to the hydrophobic nature of the organic cation.^27,28^ The increased formation and lifetime of the H-bond network would facilitate proton shuttling to and from the electrode surface, thereby promoting HER kinetics. However, neither the effect of the structure sensitivity nor the nature of the adsorption of TAA^+^ on the Pt electrode surface have been investigated.

Previous work has shown that the adsorption and organization of these organic cations at the interface are of great complexity.^24,29,30^ On Hg electrodes, the presence of needle-like capacitance peaks was associated with the existence of an interfacial film involving anion coadsorption, suggesting the adsorption of tetrabutylammonium cations (TBA^+^) as ion pairs such as Bu_4_NX or (Bu_4_N)_2_X^+^.^24^ Regarding the adsorption dynamics of TBA^+^, it was observed that the adsorption is limited by the slow diffusion of the TBA^+^ to the electrode surface and that the formation of a two-dimensional adsorption layer takes place. The study revealed that the formation of the two-dimensional adsorption layer is highly influenced by the composition and concentration of the background electrolyte.^29^ This level of complexity is also expected to occur for TBA^+^ adsorption on other electrode materials, such as Pt.

To unravel the nature of TBA^+^ adsorption on Pt electrodes and correlate it with its effect on the increased HER activity in alkaline media, we present here a systematic approach of the adsorption of TBA^+^ on Pt single-crystal electrodes. The findings obtained in this study indicate that the highest HER activity is associated with the formation of a dynamic, reversible, two-dimensional adsorption layer of TBA^+^. Physisorbed TBA^+^ obstructs active sites on the electrode surface, leading to a reduction in the coverage of underpotential-deposited (UPD) H*. However, a simultaneous remarkable enhancement of the HER activity is observed in the potential window usually ascribed to overpotential-deposited (OPD) H*. The results outlined in this article illustrate and partially clarify the complexities of TBA^+^ accumulation on Pt single-crystal electrodes and provide new molecular-level insights for optimizing the HER on different atomic-level electrode geometries.

## Experimental Section

The electrochemical cell was cleaned by immersing it overnight in a 20 mM solution of KMnO_4_ (≥99.0%). The pH of the solution was set to ≤1 with H_2_SO_4_ (96%). All of the material employed was rinsed with a freshly prepared 10% mixture of H_2_SO_4_ and H_2_O_2_ followed by repetitive rinsing and boiling in ultrapure water (Milli-Q, 18.2 MΩ cm).

### Cyclic Voltammetry (CV)

Cyclic voltammetry was performed by using a standard three-electrode cell assembly with a BioLogic VSP300 potentiostat. Pt wire was used as the counter electrode and a HydroFlex reference electrode, connected with an additional 10 μF shunt capacitor. All the potentials in this article are reported vs the reversible hydrogen electrode (RHE) scale. Pt(111) and Pt(553) 4 mm-diameter electrodes (99.999%) were used as working electrodes and prepared according to the flame annealing method reported by Clavilier.^31^ Single-crystal electrodes were cooled in an Ar/H_2_ (1:3) environment to obtain a well-ordered structure.

NaOH solution (30% Suprapur) and TBAOH (40 wt % in H_2_O Sigma-Aldrich) were used as electrolytes (pH 12). TBAClO_4_ (≥99.0%, Sigma-Aldrich) and NaClO_4_ (≥98.0%, Sigma-Aldrich) were used for salt additions. The electrolyte was purged with Ar. Current densities (*j*) were calculated by normalizing the current to the geometric area of the Pt electrode (12.566 mm^2^), which for single-crystal electrodes corresponds to the electrochemically active surface area.

### CO Displacement Experiments

CO was dosed inside the Ar-purged electrolyte for the charge displacement experiments at a given constant potential at which CO is readily adsorbed on the Pt single-crystal surface. The transient currents required to displace the adsorbed species to maintain the set potential were recorded. The theoretical details of the charge displacement using CO are explained elsewhere.^32,33^ The electrolyte was replaced frequently during charge displacement experiments to avoid contamination of the electrolyte with carbonates that form when CO is introduced in alkaline medium.

### In Situ Shell-Isolated Nanoparticle-Enhanced Raman Spectroscopy (SHINERS)

The synthesis of SiO_2_-coated Au nanoparticles (Au@SiO_2_ NPs) for SHINERS experiments was carried out following the protocol described by Tian et al.^34,35^ 2.4 mL of 1% HAuCl_4_ (99.9% Sigma-Aldrich) was added to 200 mL of ultrapure water (Milli-Q, 18.2 MΩ). The resulting solution was refluxed to 90 °C under vigorous stirring. 1.5 mL of 1% sodium citrate (99.0% Sigma-Aldrich) was added to the solution of HAuCl_4_ to obtain Au NPs of approximately 55 nm diameter. 400 μL of 1 mM (3-aminopropyl)trimethoxysilane (APTMS) (97% Sigma-Aldrich) was added dropwise to 30 mL of the dispersion of Au NPs. The solution was placed under vigorous stirring for 20 min at room temperature. 3.2 mL of 0.54% sodium silicate solution (27% SiO_2_ Honeywell) at pH 10.5 was added and allowed to stir for another 3 min. The dispersion was transferred into a 98 °C water bath under stirring for 30 min. Finally, the Au@SiO_2_ NP suspension was quickly cooled in an ice bath, centrifuged three times, and diluted with ultrapure water. Au@SiO_2_ NPs were drop-cast onto the Pt(111) single crystal and dried under Ar for in situ Raman measurements. Before any Raman spectra were taken, a potential of −0.2 V in NaOH pH 12 was applied to the Pt(111) electrode covered in Au@SiO_2_ NPs to clean the surface. The coverage of the Au@SiO_2_ NPs was estimated to be <30%.^36^

In order to ensure the suitability of Au@SiO_2_ NPs for SHINERS, pinhole and enhancement tests were performed according to the protocol provided by Tian et al.^34,35^ For Raman pinhole tests, 5 μL of Au@SiO_2_ NPs was deposited onto a glassy carbon substrate by drop casting. 2 μL of a 10 mM pyridine (99.8%, Sigma-Aldrich) solution was dropped on top of the deposited Au@SiO_2_ NPs. Enhancement tests were similarly performed but using a gold substrate instead.

Raman spectra were recorded with an HR-800 (Jobin Yvon-Horiba) Raman spectrometer integrated with a confocal microscope. The spectra were obtained by excitation with a 17 mW He–Ne laser with a wavelength of 632.8 nm. All the Raman measurements were performed in a spectro-electrochemical cell, and the electrolyte was kept oxygen-free by Ar bubbling above the electrolyte while taking the spectra. The potential was applied for 20 s before the data collection, and the averaging time used in the collection of spectra was 10 s. The spectra given in this article were reference-subtracted. The reference used was a spectrum of Pt(111) in NaOH at pH 12 in the double-layer region at 0.45 V, where no specific adsorption takes place.

All cyclic voltammetry and CO displacement experiments were repeated three and four times, respectively. Replicates were performed on different days with a clean cell and a fresh electrolyte each day. For SHINERS replicates, the experiment was performed twice, on different days but using the same batch of Au@SiO_2_ NPs.

## Results and Discussion

**Figure 1:**
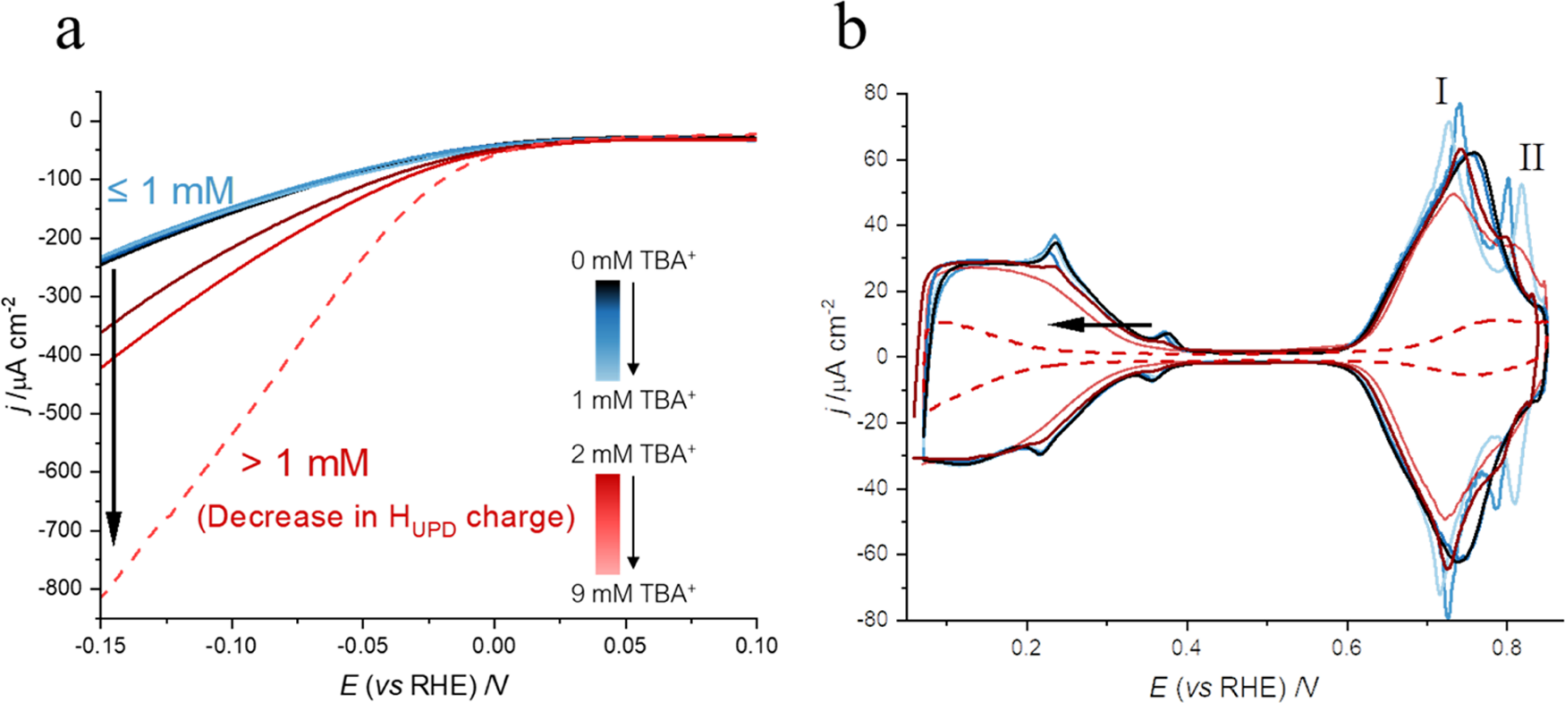
(a) Linear sweep voltammogram of Pt(111) in NaOH pH 12 (solid) with TBAClO_4_ of 0–1 mM (blue) and 2–5 mM (red) and in TBAOH pH 12 (dashed) during the HER. The HER currents are normalized by the geometrical area of the electrode. The labels indicate that at concentrations of TBA^+^ above 1 mM, the H_UPD_ charge of the voltammogram decreases compared to that of the blank in NaOH. (b) Cyclic voltammogram of Pt(111) NaOH pH 12 (solid) with TBAClO_4_ 0–1 mM (blue) and 2–5 mM (red) and in TBAOH pH 12 (dashed). Scan rate: 50 mV s^–1^.

Tetraalkylammonium cations (TAA^+^) have been shown to lead to a substantial enhancement of the HER kinetics in alkaline media on polycrystalline Pt electrodes.^25^Figure 1 illustrates that for Pt(111), the effect of the organic tetrabutylammonium cation (TBA^+^) on the activity of the alkaline HER strongly depends on its concentration. Low concentrations of TBA^+^ (TBAClO_4_ ≤ 1 mM, NaOH pH 12 background electrolyte) present no effect on the HER currents (Figure 1a, blue lines) and on the H_UPD_ (Figure 1b, blue lines). However, a splitting of the broad peak corresponding to OH_ads_ at 0.72 V is observed in the presence of TBA^+^ ≤ 1 mM (peaks I and II in Figure 1b). Peak I shifts toward less positive potentials with peak II shifting to more positive potentials with increasing concentrations of TBA^+^ (replicates are shown in Figure S1). Splitting of the OH_ads_ peak in alkaline media has also been observed for Li-containing electrolytes.^37^

While the nature of these two peaks has not yet been resolved, we suggest that it originates from the different interaction or coadsorption of TBA^+^ and Na^+^ with the OH_ads_ adlayer. The organic cation is highly hydrophobic^27^ and cannot form favorable interactions with H_2_O, which forces adjacent H_2_O molecules to reorganize. In fact, it has been shown that the hydrophobicity of these cations effectively impedes the irreversible oxidation and surface roughening of Pt(111) electrocatalysts during oxide formation and reduction processes in alkaline media by inhibiting the formation of OH_ads_/O_ads_(H_2_O) on the surface.^38^ On the other hand, Na^+^ establishes stronger interactions with H_2_O in its solvation shell than TBA^+^.^27,39^ Different interactions with H_2_O could alter the state of the OH_ads_ adlayer on Pt(111). Importantly, these results show that at these low concentrations, TBA^+^ does certainly interact with the surface, although this is not visible from the H_UPD_ region. The total charge in both the H_UPD_ region (0.07–0.40 V) and the OH_ads_ region (0.60–0.85 V) remains unchanged (Table S1), implying the same H_UPD_ and OH_ads_ coverage, which discards any contamination or specific adsorption of TBA^+^ ≤ 1 mM on Pt(111) electrodes.

On the other hand, at concentrations of >1 mM (red lines in Figure 1), a decrease in charge in both the H_UPD_ and OH_ads_ regions is observed, exclusively caused by the effect of the TBA^+^ and not by the anion (see Figure S2). Remarkably, an increase in current during the HER is observed solely when there is a concurrent drop in the overall charge of the CV, attributed to the accumulation of TBA^+^ at the interface. In agreement with this observation, the highest HER currents occur at the highest concentration of TBA^+^ used in this study (9 mM TBAOH, dashed line). The maximum interfacial excess of TBA^+^ was achieved by using TBAOH instead of a mixture of NaOH and TBAClO_4_, due to the very low solubility of TBAClO_4_ in water.^40^

The decrease in charge observed in the H_UPD_ and OH_ads_ regions in the CV, induced by high concentrations of TBA^+^, has previously been assigned to site blocking.^25,41^ While site blocking typically inhibits the HER and diminishes the H_UPD_ coverage on the surface,^42^ TBA^+^ is an unusual site-blocking element in the sense that instead, it enhances the HER activity in alkaline media. This phenomenon of lowering H_UPD_ coverage has been employed to re-evaluate the effective surface area through the H_UPD_ charge, leading to an increased effective HER activity in the presence of TAA^+^.^25^ However, in the same study, the chemisorption of TAA^+^ on Pt electrodes was discarded,^25,43^ and therefore, the H_UPD_ charge should be considered to give an inaccurate estimate of the effective surface area. Furthermore, both the adsorption kinetics and the location of TBA^+^ at the interface have not yet been described for Pt, and Figure 1 clearly shows an unexpected concentration dependence.

**Figure 2:**
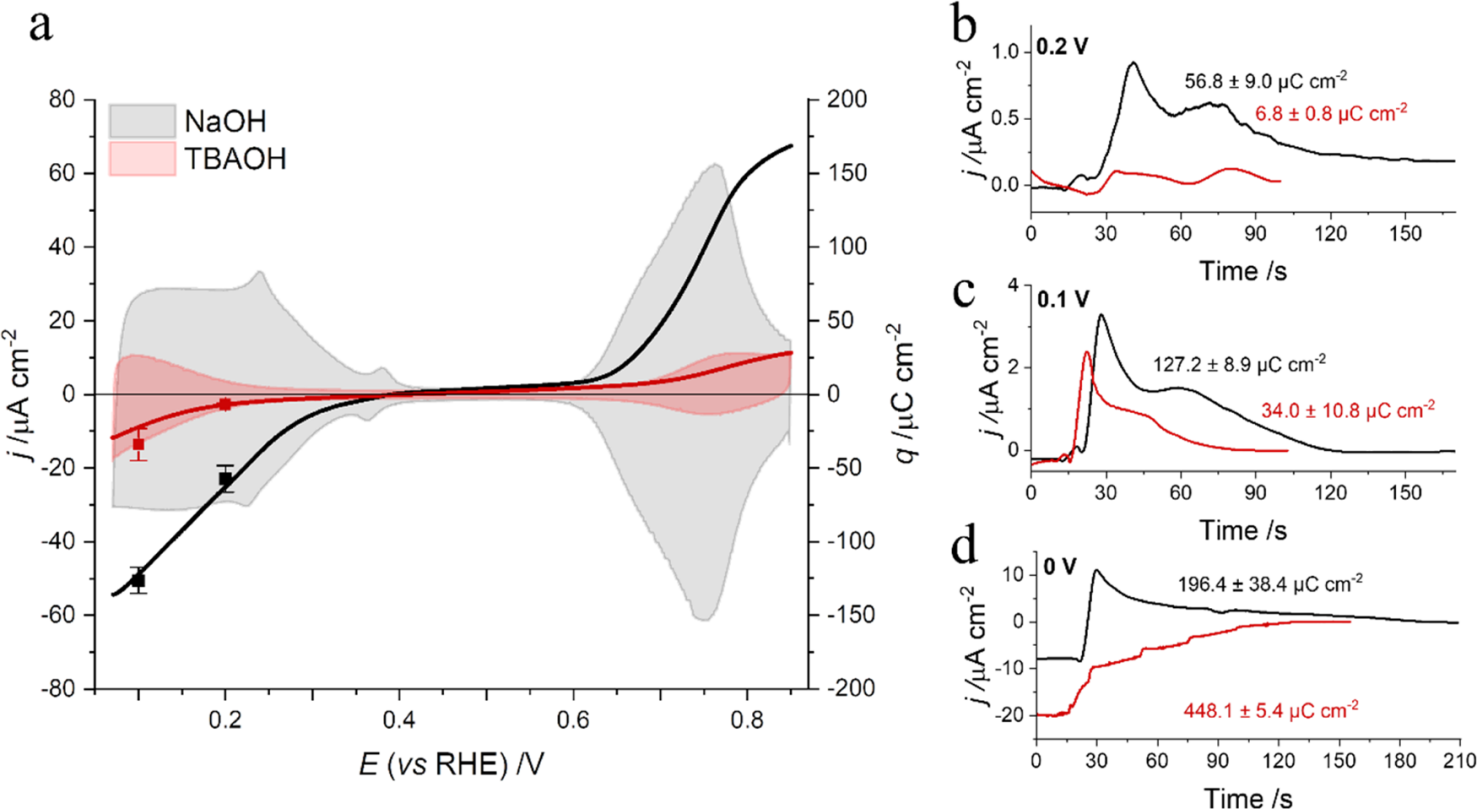
(a) Cyclic voltammogram and total charge curve of Pt(111) as a function of potential in NaOH (black) and TBAOH (red) at pH 12. Scan rate: 50 mV s^–1^. The left ordinate of the cyclic voltammogram is the current, and the right ordinate represents the total charge. The bars depict the standard deviation of the charge displacement, calculated by averaging four values obtained on four separate days when the experiment was replicated. (b) Current transient of the CO displacement of Pt(111) in NaOH (black) and TBAOH (red) pH 12 at 0.1 V. (c) Current transient of the CO displacement of Pt(111) in NaOH (black) and TBAOH (red) pH 12 at 0.2 V. (d) Current transient of the CO displacement of Pt(111) in NaOH (black) and TBAOH (red) pH 12 at 0 V.

The chemisorption of TAA^+^ on Pt electrodes has been regarded as unlikely due to the chemical inertness of the alkyl groups.^25,43^ To confirm the absence of the chemisorption of TBA^+^, we performed charge displacement experiments. During charge displacement, CO is dosed into the electrochemical cell at a constant potential, displacing the initial surface-adsorbed species (Figure S3). The transient current recorded during the forced desorption provides the displaced charge. Positive current transients during CO displacement indicate a negative total surface charge before CO introduction. By conducting displacement experiments at different potentials, a charge versus potential curve can be constructed, aiding in determining the potential of zero total charge (pztc). Figure 2a shows that the same values of pztc are measured in the absence and presence of TBA^+^, discarding the chemisorption of TBA^+^ on the Pt(111) surfaces. The total charge curves in Figure 2a were obtained from the integrated voltammetric profile, using the charge displaced with CO as the integration constant. A description of the theoretical background and the details of the procedure used to obtain these values can be found in the literature.^32,44^ The uncorrected pztc of a well-ordered Pt(111) at pH 12 measured in NaOH is 0.39 V (−0.32 V vs standard hydrogen electrode (SHE)), which correlates well with the literature (−0.326 V vs SHE at pH 12.3).^45^ Below the pztc, the transient current is positive, which agrees with the oxidative desorption of H_UPD_ (Figure 2b,c).^32,33,45−47^ The value of the pztc of Pt(111) in the presence of TBA^+^ is the same as for NaOH, which confirms the lack of chemisorption of the organic cation. Nevertheless, a decrease in the total charge curve and in the transient currents is observed in the H_UPD_ region (Table S2), indicating that despite the lack of chemisorption, the presence of TBA^+^ decreases the H_UPD_ coverage on the Pt surface. Furthermore, the effect of TBA^+^ on the HER can also be observed in Figure 2d, where a background HER current is detected in the charge displacement at 0 V, which is not observed in the absence of TBA^+^.

**Figure 3:**
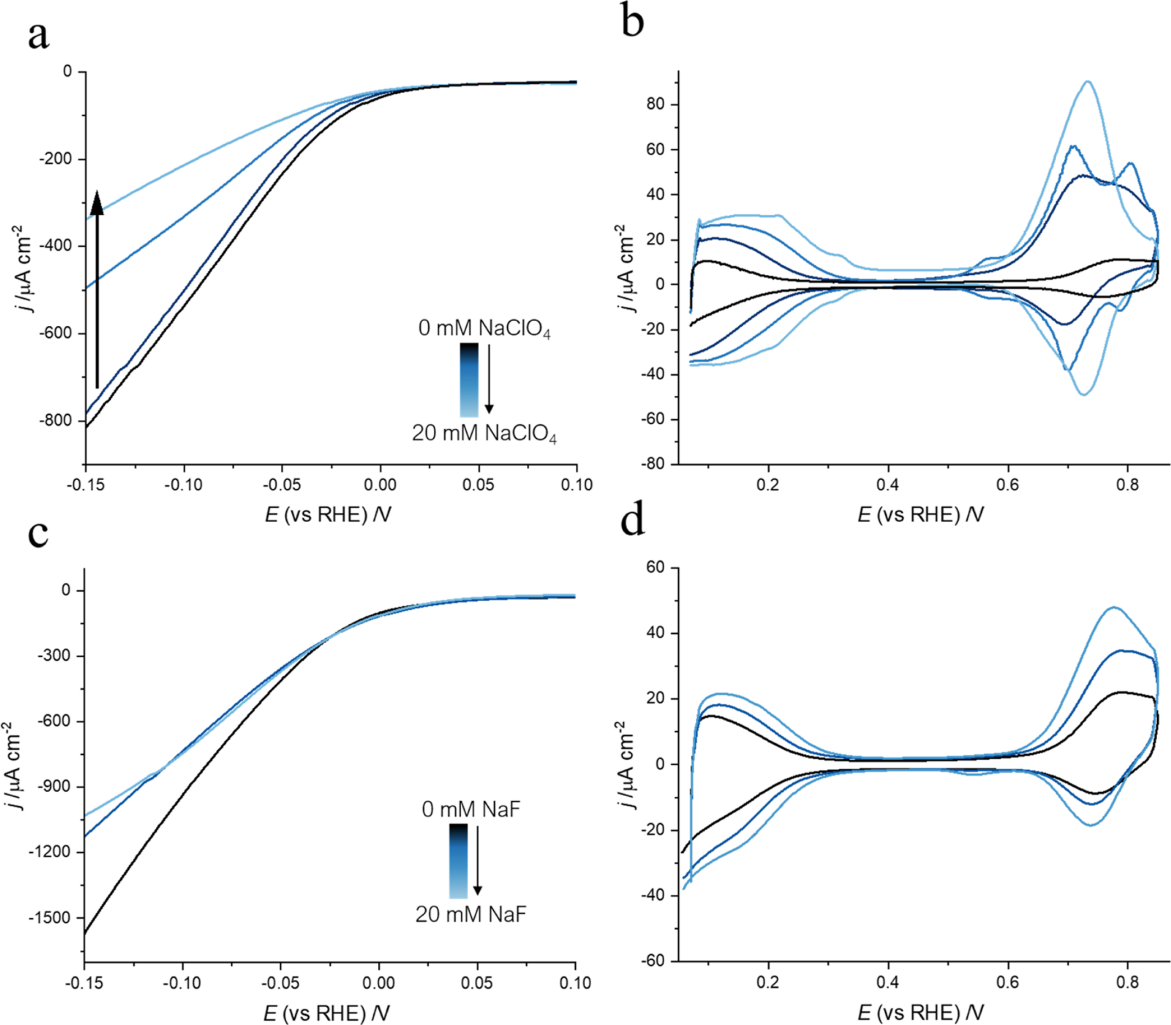
(a) Linear sweep voltammogram of Pt(111) in TBAOH pH 12 (black) with NaClO_4_ 0–10 mM (different shades of blue) pH 12 during the HER. Scan rate: 50 mV s^–1^. The HER currents are normalized by the geometrical area of the electrode. (b) Cyclic voltammogram of Pt(111) TBAOH pH 12 (black) with NaClO_4_ of 0–20 mM (different shades of blue). Scan rate: 50 mV s^–1^. (c) Linear sweep voltammogram of Pt(111) in TBAOH pH 12 (black) with NaF 0–20 mM (blue) during the HER. (d) Cyclic voltammogram of Pt(111) in TBAOH pH 12 (black) with NaF 0–20 mM (blue). Scan rate: 50 mV s^–1^. A partial recovery of the surface is observed when Na^+^ salts are added to the electrolyte, which demonstrates that the accumulation of TBA^+^ in the diffuse layer contributes to the apparent site blocking of the surface. Physisorbed TBA^+^ on the surface is not completely removed with the addition of NaF. The effect of 20 mM NaClO_4_ is more evident than that of 20 mM NaF due to the low solubility of TBAClO_4_ that precipitates in solution when NaClO_4_ is added.

By dismissing the chemisorption of TBA^+^, the decrease in the H_UPD_ charge and the apparent site blocking observed when TBA^+^ accumulates at the interface should, therefore, be explained by the physisorption of the cation on the metal surface. Interestingly, the physisorption of TBA^+^ depends on the concentration of the organic cation but also presents a slow kinetic component related to its accumulation in the diffuse double layer (DL) (see the effect of the CV cycle number illustrated in Figure S4). This complexity resembles the observations for TBA^+^ adsorption on Hg and Bi electrodes, for which it has been suggested that a condensed film of TBA^+^ forms. The film is unstable at negative potential (−0.15 V), as the HER activity decreases with time, accompanied by a partial recovery of the peaks in the blank CV (see Figure S5).^30,48^ To further demonstrate that TBA^+^ is physisorbed and resides in the diffuse double layer,^43^ we increased the concentration of a nonadsorbing, inert salt.^49^ A decrease in HER activity was detected when NaClO_4_ was added to a TBAOH solution (pH 12) (Figure 3a). Furthermore, a recovery of the H_UPD_ and OH_ads_ peaks in the blank CV of Pt(111) was observed with higher concentrations of NaClO_4_ (Figure 3b). This provides strong evidence that the organic cation resides in the diffuse layer. This effect also occurs for other nonadsorbing electrolytes (Figure 3c,d) and for other surfaces (see Figure S6). However, the anion seems to play a significant role in the removal of TBA^+^ from the interface. This can be clarified by considering both the concentration effect and the high stability of TBAClO_4_ that precipitates in solution when NaClO_4_ is added. Higher concentrations of Na^+^ vs TBA^+^ result in the disruption of the TBA^+^ film. However, when NaF is introduced in the solution, the partial recovery of the HER activity (Figure 3c) and H_UPD_ and OH_ads_ peaks (Figure 3d) is less evident compared to when NaClO_4_ is used. Because TBA^+^ has weak adsorption properties, we only witness a partial restoration of the peaks when the concentration of Na^+^ surpasses that of TBA^+^. This effect is enhanced with the addition of NaClO_4_ due to the highly stable TBAClO_4_ that presents a low solubility in water. Therefore, when NaClO_4_ is added, part of the TBA^+^ forms TBAClO_4_ that precipitates in the solution, leading to more TBA^+^ being removed from the interface and resulting in a higher recovery of the peaks.

The decrease in charge and, consequently, the increased HER rates in Pt(111) result from the physisorption of TBA^+^ on the electrode surface. The coverage of the TBA^+^ physisorbed on the surface, however, is dictated by the gradual accumulation of the organic cation in the diffuse layer. Upon reaching the maximum interfacial excess of TBA^+^, denoting the highest accumulation of the cation in the electric double layer, the peak of the HER activity occurs.

**Figure 4:**
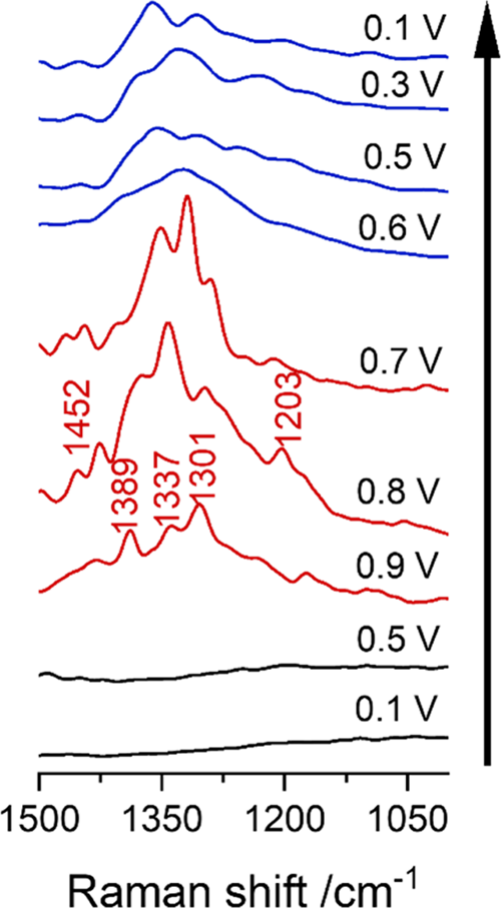
Potential dependent in situ SHINERS spectra of Pt(111) in TBAOH pH 12. The Raman bands corresponding to the vibrational modes of TBA^+^ are labeled in the spectra. The arrow indicates the order in which the measurements were taken.

Shell-isolated nanoparticle-enhanced Raman spectroscopy (SHINERS) was used to confirm the accumulation of TBA^+^ at the Pt(111) interface as a function of potential (Figure 4). The lack of bands in the first spectra taken at 0.1 and 0.5 V (black lines) confirms that the accumulation of TBA^+^ at the interface is limited by the slow kinetics of the organic cation approaching the electrode surface. At 0.9 V, bands within the 1200–1500 cm^–1^ range, associated with the C–H vibrational modes of TBA^+^, arise. The intensity of these bands increases as the potential is reduced to 0.8 and 0.7 V, remaining within the OH_ads_ region. This observation confirms the results obtained by CV that there is a distinct interaction between TBA^+^ and OH_ads_. A decrease in the intensity of the Raman bands corresponding to TBA^+^ is observed when the potential is reduced into the H_UPD_ region. The intensity of these bands remains stable for the spectra taken in the double layer (DL) (0.6–0.5 V) and H_UPD_ (0.3–0.1 V), suggesting that once the film of TBA^+^ is formed, it remains stable. The results obtained with SHINERS prove that there is an interaction between TBA^+^ and the Pt surface, especially when the Pt surface is covered with OH_ads_.

**Figure 5:**
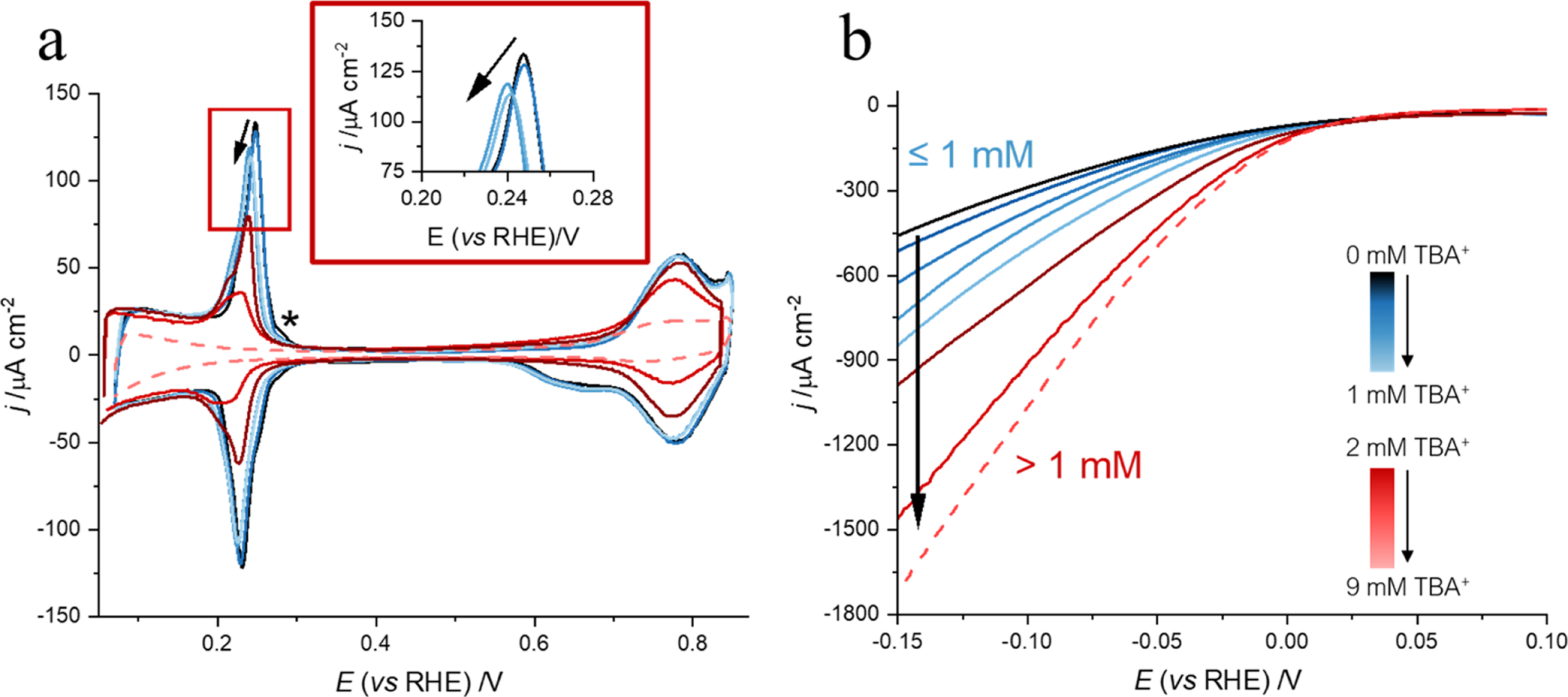
(a) Cyclic voltammogram of Pt(553) NaOH pH 12 (solid) with TBAClO_4_ of 0–1 mM (blue) and 2–5 mM (red) and in TBAOH pH 12 (dashed). (b) Linear sweep voltammogram of Pt(553) in NaOH pH 12 (solid) with TBAClO_4_ 0–1 mM (blue) and 2–5 mM (red) and in TBAOH pH 12 (dashed) during the HER. Scan rate: 50 mV s^–1^. The HER currents are normalized by the geometrical area of the electrode.

Another factor that affects the accumulation of TBA^+^ at the interface is the geometric structure of the electrode surface. To demonstrate this, we study the effect of TBA^+^ on HER activity on a Pt(553) electrode, whose nominal structure is formed by {111} five atom-wide terraces separated by {110} monatomic steps. Pt(553) can display “step bunching”, observable by the small shoulder in the step-related peak (represented with * in Figure 5a).^50^ In the presence of a low concentration of TBA^+^ (≤1 mM) (blue lines), in a pH 12 NaOH background electrolyte, the CV of Pt(553) exhibits small but reproducible differences compared to the blank voltammogram in NaOH. A small decrease in the charge of the H_UPD_ region (between 0.07 and 0.30 V) is observed (Table S3), mainly related to the step-related peak, also accompanied by the disappearance of the peak corresponding to step bunching. The step-related peak at 0.25 V, which corresponds to the displacement of H_UPD_ with OH_ads_ at the steps,^16,44,51,52^ shifts to less positive potentials as the concentration of TBA^+^ increases. This shift has also been observed with alkali metal cations and has been ascribed to an interaction between the cation and (the interfacial water solvating) the OH_ads_.^9,16,37^ In contrast to Pt(111), this low concentration of TBA^+^ leads to an enhancement of HER activity, which must be related to the presence of the following steps. At higher concentration of the TBA^+^ (>1 mM), the H_UPD_ and OH_ads_ charges corresponding to the {111} terrace sites decrease and the HER activity continues to increase, similarly to Pt(111). Therefore, step sites enhance the effect of TBA^+^, with HER enhancement at lower concentrations of TBA^+^ (<1 mM) and much higher values of currents measured during the HER for Pt(553) than for Pt(111) (Figure 5b).

We have not studied the reverse reaction, i.e., hydrogen oxidation reaction (HOR), but since the HER and HOR on Pt are quasi-reversible, it has been found that electrolyte effects of the HER and HOR on Pt single-crystal electrodes are identical.^53^ We would expect the same for the effect of the organic films.

## Conclusions

The addition of tetrabutylammonium (TBA^+^) to the electrolyte is associated with an increase in HER activity on Pt single-crystal electrodes in alkaline media. The concentration of the organic cation plays a crucial role in influencing the activity of the alkaline HER on Pt(111). At low concentrations (≤1 mM), no significant effect is observed on either the HER currents or the coverage of H_UPD_. However, a splitting of the broad peak corresponding to OH_ads_ is noted, suggesting a distinct interaction between the TBA^+^ and OH_ads_, as confirmed by in situ Raman spectroscopy. At concentrations of TBA^+^ > 1 mM, a decrease in charge in both the H_UPD_ and OH_ads_ regions is detected, indicating an apparent site blocking effect that leads to a counterintuitive increase in HER activity. The increased HER is determined by the interfacial excess of TBA^+^, which depends on both its concentration in the electrolyte and the kinetics of its accumulation at the interface. In spite of the observed apparent site blocking, TBA^+^ does not chemisorb on the Pt(111) surface. Rather, it forms a physisorbed layer or film (at sufficiently high concentration), which can be removed by replacement with an inert electrolyte or by applying very negative potentials. This physisorbed film appears to be reminiscent of the condensed TBA^+^ film reported previously for Hg and Bi electrodes. Apparently, this film lowers the H_UPD_ coverage of the surface in the UPD region but enhances the HER in the OPD region. In the presence of steps on the Pt surface, the effect of TBA^+^ is enhanced with respect to Pt(111). TBA^+^ appears to “block” step sites, as manifested by changes in the step-related peaks, but it enhances the HER, for all TBA^+^ concentrations.

These results highlight the highly complex behavior of TBA^+^ at the electrochemical interface. It remains to be determined if “hydrophobicity” is the key or only feature of these films in enhancing the HER. The counterintuitive effect of the TBA^+^ film on hydrogen UPD vs HER activity is an unsolved puzzle. Future efforts should focus on understanding the kinetics and the detailed properties of the TBA^+^ film and establishing the exact molecular mechanism through which it influences hydrogen adsorption and hydrogen evolution.

## Supporting Information Available

Replicates, values measured in the charge analyses, blanks to test the CO coverage after charge displacement experiments, and additional cyclic and linear voltammetry data (PDF)
