# Supplementary material for: Effect of a Physisorbed Tetrabutylammonium Cation Film on Alkaline Hydrogen Evolution Reaction on Pt Single-Crystal Electrodes
Source: ACS Catal. 2024 May 9;14(11):8130–7. doi: 10.1021/acscatal.4c01765 (PMC11165451; doi:10.1021/acscatal.4c01765)
Supplement: Supplementary file 1 — cs4c01765_si_001.pdf [file cs4c01765_si_001.pdf]

Supplementary Information

Effect of a Physisorbed Tetrabutylammonium Cation Film on Alkaline Hydrogen Evolution Reaction on Pt Single-Crystal Electrodes

Julia Fernández-Vidal,[a] Marc T.M. Koper[a*]

[a]Leiden Institute of Chemistry, Leiden University, PO Box 9502, 2300 RA Leiden, The Netherlands

*Corresponding author. Email: m.koper@lic.leidenuniv.nl

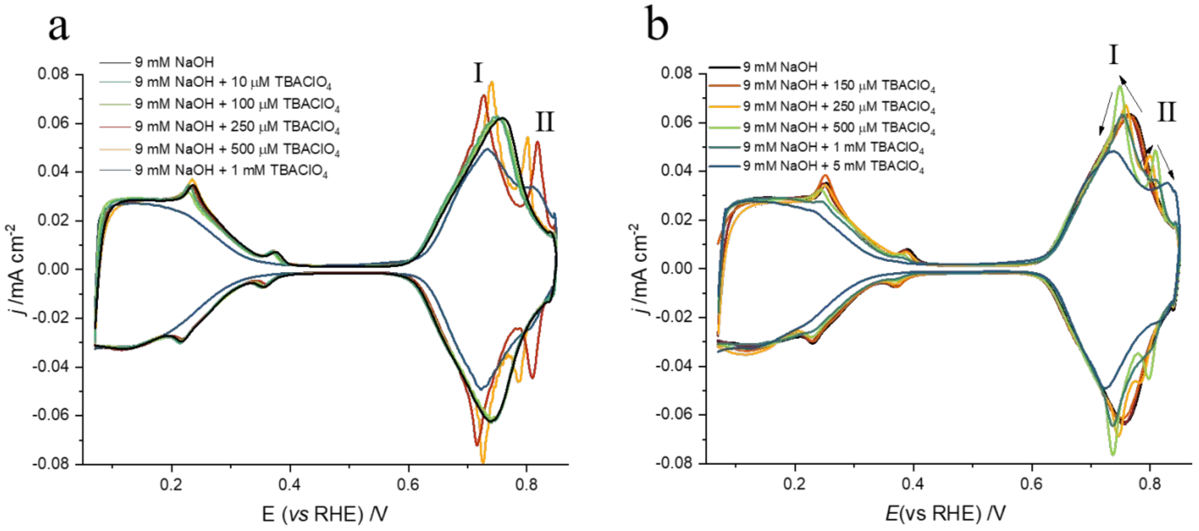

Figure S1. Replicates of Figure 1 in the main text. Cyclic voltammograms of Pt(111) in NaOH pH 12 with $TBAClO_4$ (0-5 mM). Scan rate 50 mV s^{-1}. While the same concentrations of TBA^+ in the electrolyte does not ensure the same interfacial excess of TBA^+, the progression of the peaks with increased concentrations of TBA^+ follows the same trend in all the replicates.

Table S1. Pt(111) in NaOH pH 12 with $TBAClO_4$ (0-1 mM). The charge in the H_{UPD} and OH_{ads} region remains unchanged as the concentration of TBA^+ increases which discards any site blocking for concentrations ≤ 1 mM.

TBA concentration	Charge (0.07-0.3 V)	Charge (0.6-0.85 V)	HER current at 0.15 V
0.00 mM	139.3 μC cm^{-2}	160.8 μC cm^{-2}	-269.6 μC cm^{-2}

0.10 mM	137.7 μC cm^{-2}	161.5 μC cm^{-2}	-264.1 μC cm^{-2}
0.25 mM	138.5 μC cm^{-2}	160.8 μC cm^{-2}	-257.1 μC cm^{-2}
0.50 mM	137.9 μC cm^{-2}	161.5 μC cm^{-2}	-260.4 μC cm^{-2}
1.00 mM	137.9 μC cm^{-2}	161.5 μC cm^{-2}	-256.7 μC cm^{-2}

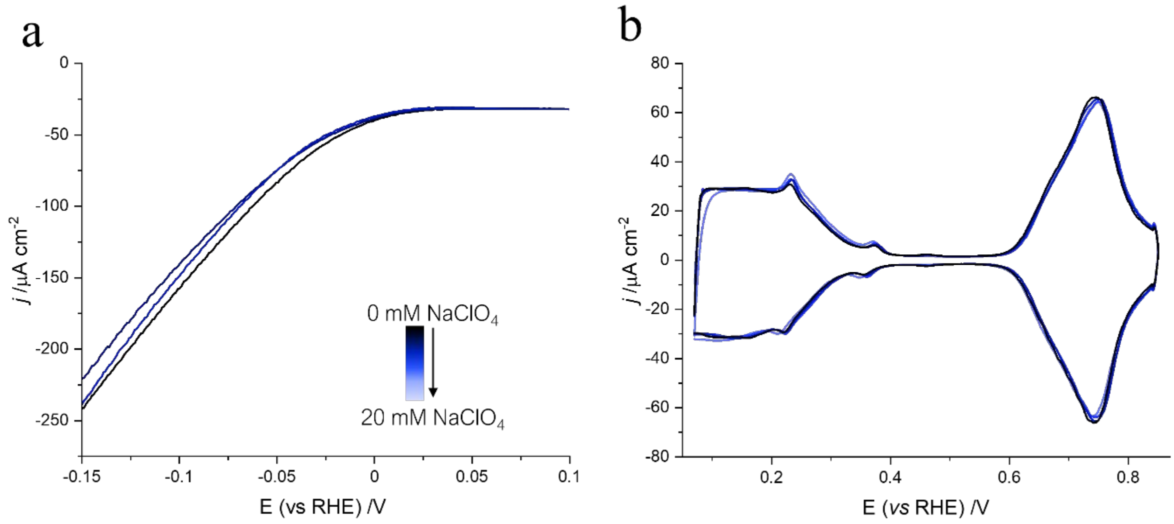

Figure S2. (a) Linear sweep voltammogram of Pt(111) in NaOH pH 12 with $NaClO_4$ (0-20 mM) during HER. (b) Cyclic voltammogram of Pt(111) in NaOH pH 12 with $NaClO_4$ (0-20 mM). Scan rate 50 mV s^{-1}. Both the HER rates and the CV profile remain unchanged when increasing the concentration of the $NaClO_4$ supporting salt, which demonstrates that the increase in HER currents upon $TBAClO_4$ addition is due to the TBA^+ and not due to the anion (ClO_4^-).

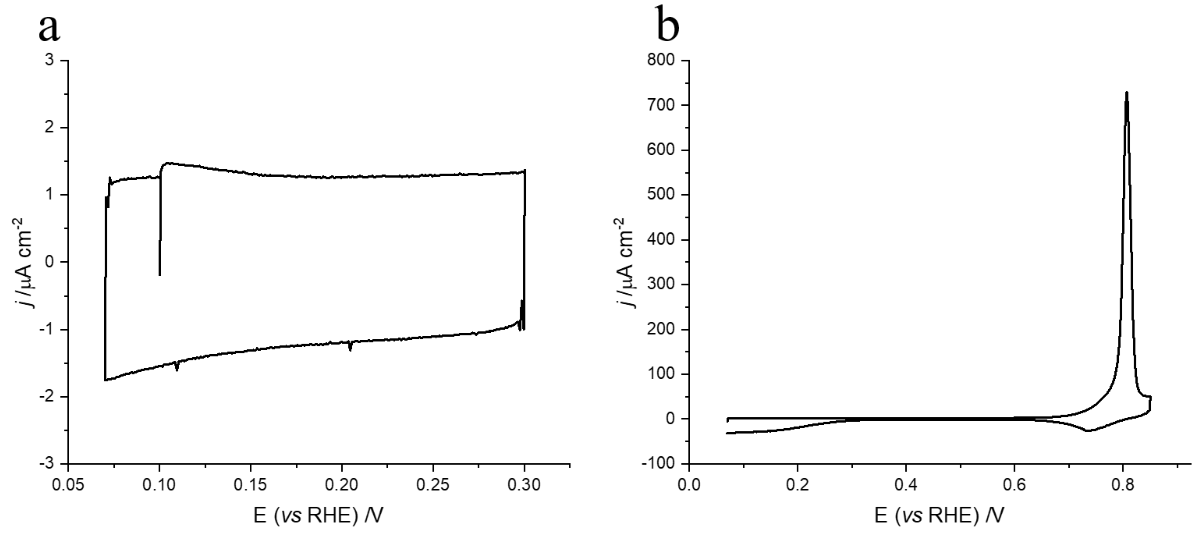

Figure S3. (a) Cyclic voltammogram of Pt(111) in TBAOH pH 12 after charge displacement experiment with CO. Scan rate 50 mV s^{-1}. (b) Cyclic voltammogram of Pt(111) in TBAOH pH 12 corresponding to the CO stripping. Scan rate 50 mV s^{-1}. In (a), the capacitive profile that the Pt(111) presents in the H_{UPD} region demonstrates that the CO is able to displace the TBA^+ from the surface and that the surface of Pt is fully covered with CO after the charge displacement experiments. This can also be observed by the charge displaced which reaches a value of zero (main text, Figure 2b-d) when the surface is fully covered with CO.

Table S2. Integrated transient currents measured in the charge displacement experiments with CO. A decrease in the transient current is observed in the H_{UPD} region, dependent on the concentration of TBA^+ which confirms that there is a decrease in the H_{UPD} coverage in the presence of TBA^+. On the other hand, an increase in the transient current occurs during HER due to a background HER current that occurs already at 0 V in the presence of TBA^+.

**E /V**	**9 mM NaOH**	**9 mM NaOH + 5 mM $TBAClO_4$**	**9mM TBAOH**
0.1	119.2 ± 8.9 μC cm^{-2}	81.6 ± 16.3 μC cm^{-2}	34.0 ± 10.8 μC cm^{-2}
0.2	43.8 ± 9.0 μC cm^{-2}	--	6.8 ± 0.8 μC cm^{-2}
0.0	196.4 ± 38.4 μC cm^{-2}	340.0 ± 185.5 μC cm^{-2}	448.1 ± 5.4 μC cm^{-2}

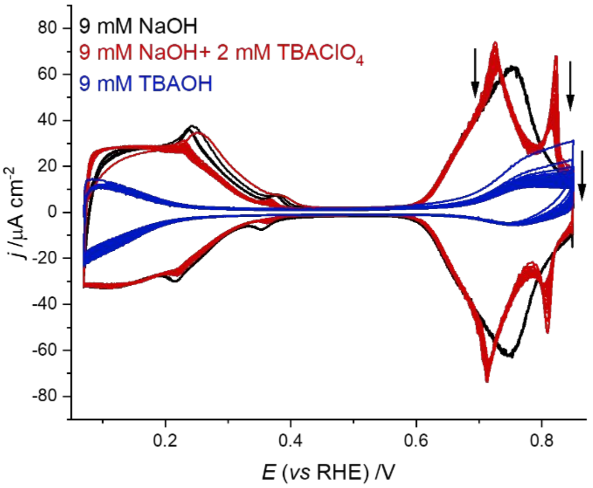

Figure S4. Cyclic voltammogram of Pt(111) pH 12 in 9 mM NaOH (black), 9 mM NaOH + 2 mM $TBAClO_4$ (red) and in 9 mM TBAOH (blue). Scan rate 50 mV s^{-1}. The arrows indicate the decrease in change observed with increasing cycle number in the presence of TBA^+. The decrease in charge indicates site blocking that occurs when the TBA^+ accumulates at the interface. The accumulation of TBA^+ on the surface depends not only on the concentration of TBA^+ used in the electrolyte, but also on the cycle number (slow kinetic effect).

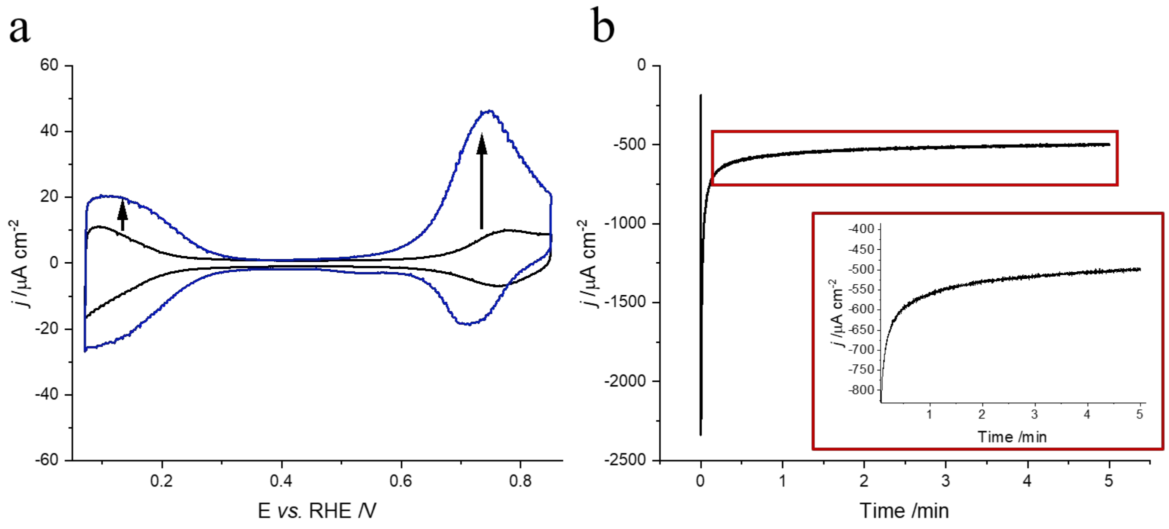

Figure S5. (a) Cyclic voltammogram of Pt(111) pH 12 in 9 mM TBAOH before (black) and after (blue) 5 minutes of HER at -0.15 V. Scan rate 50 mV s^{-1}. The arrows indicate the increase in charge observed after HER. (b) Chronoamperometry recorded during the 5 min of HER. Scan rate 50 mV s^{-1}.The partial recovery of the peaks in (a) reveal that the interfacial excess of TBA^+ decreases after HER (*i.e.* TBA^+ is removed from the surface). This can also be observed in the decrease in current during the HER in (b). The interfacial excess of TBA^+ is recovered upon cycling (see **Figure S2**).

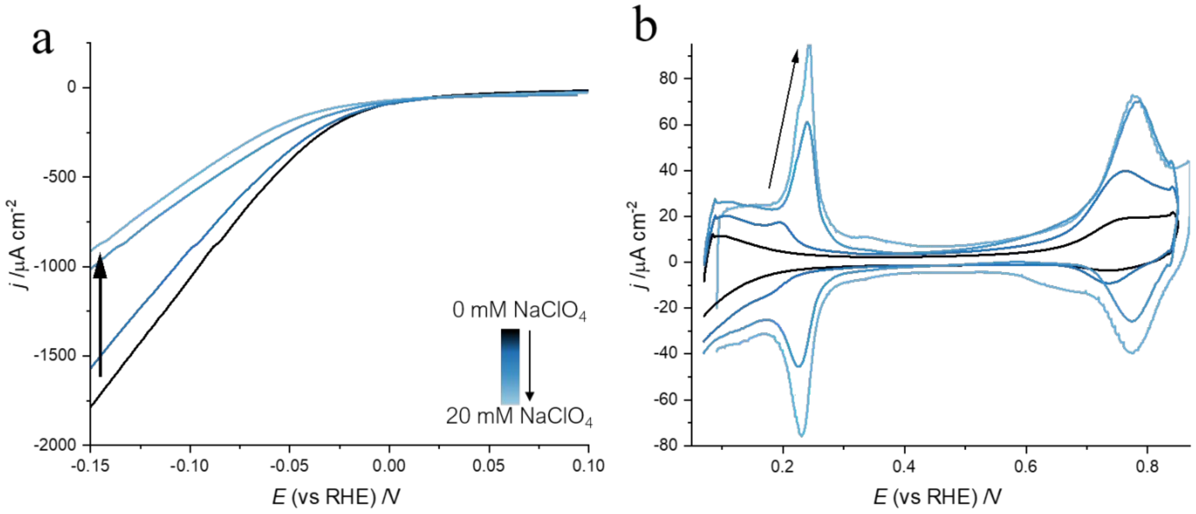

Figure S6. (a) Linear sweep voltammogram of Pt(553) in TBAOH pH 12 (black) with $NaClO_4$ 0-20 mM (blue) during HER. (b) Cyclic voltammogram of Pt(553) in TBAOH pH 12 (black) with $NaClO_4$ 0-20 mM (blue). Scan rate 50 mV s^{-1}. Further evidence that TBA^+ does not chemisorb on the surface is observed when $NaClO_4$ is added in solution. At high concentrations of TBA^+ (9 mM TBAOH), the CV shows a highly-blocked (TBA^+-saturated) surface with low charge and no defined peaks. When $NaClO_4$ is added in solution, peaks corresponding to the "clean" voltammetry of Pt(553) arise. This indicates the unblocking of active sites which demonstrates that the TBA^+ is located in the diffuse layer rather than chemisorbed on the surface. Furthermore, the HER current decreases when $NaClO_4$ is added, which supports the idea that a decrease in H_{UPD} charge occurs simultaneously with an improvement of HER activity.

Table S3. Pt(553) in NaOH pH 12 with $TBAClO_4$ (0-1 mM), showing that the charge in the H_{UPD} region decreases as the concentration of TBA^+ increases.

**TBA concentration**	**Charge (0.07-0.3 V)**	**OH_{ads} peak position**	**HER current at 0.15 V**
0.00 mM	152.8 μC cm^{-2}	0.248 V	-457.4 μA cm^{-2}
0.10 mM	150.4 μC cm^{-2}	0.247 V	-512.6 μA cm^{-2}
0.25 mM	148.8 μC cm^{-2}	0.245 V	-623.3 μA cm^{-2}
0.50 mM	146.4 μC cm^{-2}	0.241 V	-754.0 μA cm^{-2}
1.00 mM	144.0 μC cm^{-2}	0.240 V	-847.4 μA cm^{-2}
